# Remote Delivery of Partial Meal Replacement for Weight Loss in People Awaiting Arthroplasty

**DOI:** 10.3390/jcm13113227

**Published:** 2024-05-30

**Authors:** Ritesh Chimoriya, Justine Naylor, Kimberly Mitlehner, Sam Adie, Ian Harris, Anna Bell-Higgs, Naomi Brosnahan, Milan K. Piya

**Affiliations:** 1 School of Medicine, Western Sydney University, Campbelltown, NSW 2560, Australia; k.mitlehner@westernsydney.edu.au; 2 Ingham Institute for Applied Medical Research, South Western Sydney Clinical School, University of New South Wales, Sydney, NSW 2170, Australia; justine.naylor@health.nsw.gov.au (J.N.); ianaharris@unsw.edu.au (I.H.); 3 School of Clinical Medicine, University of New South Wales Medicine & Health, St George & Sutherland Clinical Campuses, Sydney, NSW 2217, Australia; sam.adie@unsw.edu.au; 4 Counterweight Limited, London W1W 7LT, UK; anna.bell-higgs@counterweight.org (A.B.-H.); naomi.brosnahan@counterweight.org (N.B.); 5 School of Medicine, Dentistry and Nursing, University of Glasgow, Glasgow G12 8QQ, UK; 6 Camden and Campbelltown Hospitals, Campbelltown, NSW 2560, Australia

**Keywords:** partial meal replacement, weight loss, arthroplasty, obesity, behaviour change

## Abstract

**Background**: Obesity is linked to higher rates of complications; lower absolute recovery of mobility, pain, and function; and increased costs of care following total knee or hip arthroplasty (TKA, THA). The aim of this prospective cohort study was to evaluate the effectiveness of a 12-week partial meal replacement (PMR) weight loss program for people awaiting TKA or THA and living with obesity (body mass index (BMI) ≥ 30 kg/m^2^). **Methods**: The intervention was delivered remotely and included a 12-week PMR plan of 1200 calories/day, incorporating two meal replacement shakes/soups and a third suitable simple meal option. The intervention support was provided through online group education sessions, one-to-one teleconsultation with a dietitian, and access to a structured PMR App with functions for goal setting and providing educational content on diet, physical activity, and behaviour changes. **Results**: Of the 182 patients approached, 29 provided consent to participate, 26 participants commenced the program, and 22 participants completed the 12-week PMR plan. Completers exhibited statistically significant weight loss from baseline to 12 weeks, with a paired difference of 6.3 kg (95% CI: 4.8, 7.7; *p* < 0.001), with 15 out of 22 (68.2%) participants achieving at least 5% weight loss. Statistically significant reductions in HbA1c and low density lipoprotein (LDL) were observed at 12 weeks compared to baseline. Moreover, a significant increase in the proportion of participants in the action and maintenance phases of the readiness to change diet, physical activity, and weight were observed at 12 weeks. The majority of program completers (18 out of 22) expressed willingness to pay for the service if offered on a long-term basis following the arthroplasty. **Conclusions**: This study’s findings demonstrated that significant weight loss is achievable for people living with obesity awaiting arthroplasty following a 12-week PMR weight loss program. The remote delivery of the intervention was feasible and well accepted by people awaiting TKA or THA.

## 1. Introduction

Obesity, defined as a body mass index (BMI) ≥ 30 kg/m^2^ [1], is a complex condition associated with chronic conditions including cardiovascular disease, type 2 diabetes, non-alcoholic fatty liver disease, and osteoarthritis [2,3,4]. Prior studies therefore suggest the importance of a chronic disease model of care to manage its associated conditions and improve health-related quality of life [2,3,4,5]. Recognising the strong association between obesity and risk of osteoarthritis as well as total arthroplasty, clinical guidelines for the management of osteoarthritis often recommend weight loss as a core strategy for people living with overweight and obesity [6,7,8,9]. For instance, the Royal Australian College of General Practitioners (RACGP) guideline for the management of knee and hip osteoarthritis strongly recommends a minimum weight loss target of 5–7.5% of body weight for people with knee and/or hip osteoarthritis as well as overweight or obesity, with greater weight loss deemed beneficial due to its association with symptomatic benefits [9]. However, an area with increasing numbers of people presenting with obesity [10], which is devoid of clear guidelines for how best to manage obesity, is total knee or hip arthroplasty (TKA, THA) [11]. Obesity is well recognised as the leading modifiable risk factor for the incidence and progression of osteoarthritis [12,13], consequently resulting in elevated rates of TKA and THA among people with obesity [14,15].

Following TKA or THA, obesity is associated with higher rates of complications including the development of deep infection and the need for any-cause revision [11,16,17], along with a lower absolute recovery in mobility as well as patient-reported pain and function [18], and increased costs of care [19,20]. Therefore, weight loss is recommended prior to surgery. Evidence from a recent randomised controlled trial demonstrated that weight loss via bariatric surgery prior to TKA reduced the risk of complications following TKA in people with severe obesity and end-stage osteoarthritis compared to those not undergoing bariatric surgery [21]. Further, 30% of participants declined TKA due to symptom improvement.

Weight loss via bariatric surgery is not a panacea, however, and bariatric surgery is not without risk [21], is costly, and not available to all. Thus, alternative methods of weight loss are required. Research suggests that significant weight loss is achievable for individuals with moderate knee osteoarthritis utilising very low energy diets, and weight loss of ≥7% is linked with significant symptom relief [22,23,24,25]. Thus, this approach may be an option for people awaiting arthroplasty. However, very low energy diets are difficult to adhere to and given people awaiting arthroplasty may prioritise their joint surgery over weight loss, the weight loss strategy may need to be easy to adhere to [26].

Partial meal replacements are often utilised for weight management [27,28], potentially balancing the necessity for rapid weight loss in individuals preparing for imminent surgery with the ease of adherence, considering the lower likelihood of acceptability or adherence to total meal replacements in a demographic not actively seeking assistance for weight loss [29]. A recent single-cohort pilot study conducted in Spain, involving 81 women with obesity awaiting TKA, reported a weight loss of 8% following a three-month partial meal replacement diet [27]. Moreover, weight loss was independently linked with improvement in index joint symptoms as well as quality of life. 

A recent systematic review indicated that while short-term, nonsurgical, preoperative weight loss interventions before TKA and THA produce statistically significant weight loss, it is not known whether the weight loss is clinically significant (defined as a 5% change in weight) or sufficient to improve outcomes after arthroplasty [30]. Recent attempts to manage obesity amongst participants undergoing pre-surgery optimisation have failed to achieve significant weight loss [31]. Clinical trials are clearly needed to inform both how weight loss is best achieved and how much weight loss is required to reduce the risks associated with obesity. Therefore, this study aimed to assess the effectiveness of a 12-week calorie-restricted partial meal replacement program for people awaiting TKA or THA and living with obesity. The primary objective of this study was to evaluate whether weight loss of 5% or higher is achievable for >50% of patients following a clinician-supported 12-week partial meal replacement weight loss program. The secondary objectives included assessment of improvements in clinical health and patient-reported outcomes, along with evaluation of the acceptability and adherence as well as the safety of the partial meal replacement weight loss program.

## 2. Methods

### 2.1. Study Design

This is a prospective pilot cohort study where participants were recruited from a waiting list of people awaiting TKA or THA in a high-volume (~600 procedures per year pre-COVID-19) public arthroplasty centre in Sydney, Australia. This study was conducted between January 2022 and August 2022, and the intervention was delivered remotely via telehealth due to COVID-19 restrictions and supported by a team of dietitians with access, if required, to a previously described publicly funded multidisciplinary weight management program [32,33,34,35]. All eligible participants were invited to participate in this study and only those who provided informed consent were included. This study was registered at the Australian New Zealand Clinical Trials Registry-ANZCTR (registration number ACTRN12621001332819). The Transparent Reporting of Evaluations with Nonrandomized Designs (TREND) checklist [36], which is frequently used for intervention evaluation studies using nonrandomized designs, was employed to ensure reporting quality of the study evaluation (Supplementary Table S1).

### 2.2. Participants

Potential participants were identified by the clinicians at their routine clinical review after being waitlisted for TKA or THA and then screened by the research team for eligibility for the study. Eligible participants were patients with a BMI ≥ 30 kg/m^2^ who were waitlisted for primary unilateral or bilateral TKA or THA for knee or hip osteoarthritis. Exclusion criteria, selected based on prior studies utilising meal replacements [28,37], included patients with a primary diagnosis other than osteoarthritis, current insulin use, diagnosis of an eating disorder, weight loss of more than 5 kg in the past three months, and current participation in another weight loss program. Patients who had undergone bariatric surgery in the previous two years were considered to be in the postoperative weight loss period and were excluded [38]. Similarly, patients unable to understand spoken/written English, and those with severe mental health problems, cognitive impairment, or conditions that could interfere with the patient’s ability to understand the study requirements were also excluded. 

### 2.3. Intervention

**Figure 1:**
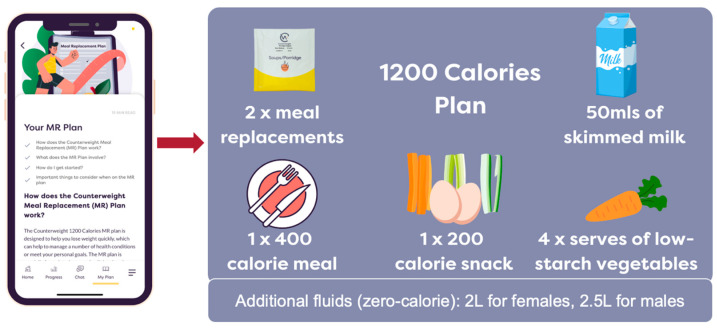
Partial meal replacement plan used in the study.

All eligible participants who agreed to participate were advised to follow a partial meal replacement (PMR) plan of 1200 calories/day for 12 weeks as outlined in Figure 1. This was supported by online education sessions, one-to-one consultation with a dietitian, and access to a structured PMR App.

#### 2.3.1. Partial Meal Replacement Plan

The PMR plan provided was 1200 calories per day (5020 kJ/day) for 12 weeks, which included meal replacement shakes or soups to cover two meals per day, and a third suitable simple meal option. The low-calorie meal replacement shakes (Example—in serving size: 54 g chocolate shake, 845 kJ energy, 3.3 g fat, 21.4 g carbohydrate and 20.1 g protein) used in this study were previously used in the DiRECT-Australia study [37,39]. These shakes/soups were provided free of cost to all participants by the research team. The timing of the meal replacement was flexible, and the participants could consume their simple meal at any time of the day. The simple meal included options for either low-carbohydrate or low-fat meals (approximately 400 calories), personalised through discussions with the dietitian, aiming for a 1200 calories intake per day for all patients.

#### 2.3.2. Supporting the Partial Meal Replacement Plan

The participants were provided with additional support throughout the study duration to follow the PMR plan. At baseline, an online one-hour group education session with 5–10 participants in each group was provided via telehealth before the initiation of the PMR. The education session focused on behaviour change and was delivered by two dietitians, and participants were provided with information and advice on the PMR and suitable meal options. Online group education sessions were also delivered at 6 and 12 weeks for an hour each, and the dietitian provided advice on long term weight loss maintenance as part of the 12-week session. The intervention was further supported by one-to-one telephone/telehealth support for 15 min with a dietitian trained in motivational interviewing at 2, 4 and 9 weeks. Participants received overall guidance on the meal replacements and portion-controlled meals at each visit, along with general advice on fluid intake and the avoidance of snacking, along with an opportunity to address any particular concerns and track their progress. The participants were also provided access to a structured PMR App, which has the following functions: tracking, goal setting, educational content (diet, physical activity, behaviour change), and one of the screenshots of the PMR App is shown in Figure 1. An endocrinologist was available to advise on any abnormal blood tests noted or clinical problems that arose during the trial.

### 2.4. Data Collection Measures

Data collection was conducted at the start of the program (baseline) and after the completion of the PMR plan (12 weeks). The data collection measures at each timepoint are summarised in Table 1.

### 2.5. Outcomes

The primary outcome of this study was to evaluate whether weight loss of 5% or higher is achievable for >50% of patients following a clinician-supported 12-week partial meal replacement weight loss program. To evaluate weight loss, weight was self-recorded by the participants at the same time of the day and reported back to the research team, with a photograph of the weight record in the weighing machine. The self-reported weight was cross validated with the images received by the research team and electronic medical records. Secondary outcomes assessed included acceptability, willingness, adherence, and safety of the intervention. Other outcomes included changes in index joint symptoms, self-reported quality of life and wellbeing, psychological distress, readiness to change lifestyle behaviour, and physical activity using the measures described in Table 1, along with improvements in clinical health outcomes and biochemical profile, including changes in the lipid profile as well as kidney and liver function.

### 2.6. Statistical Analysis

The Statistical Package for Social Sciences, Version 28 (SPSS for MacOS; SPSS Inc., Chicago, IL, USA) was used for the statistical analysis. Continuous variables are presented as mean and 95% confidence intervals (CI). The Shapiro–Wilk test was performed to examine the normal distribution of the continuous data, and the normally distributed continuous data were compared using paired t-tests. Non-parametric data were compared using Wilcoxon signed-rank tests. Mean differences in observations at two timepoints were calculated as an effect size. Similarly, categorical variables are presented using frequency and percentage and were compared using McNemar’s tests. All tests with *p*-values < 0.05 were considered statistically significant.

### 2.7. Ethics Approval

Ethics approval for this study was granted by the South Western Sydney Local Health District Human Research Ethics Committee (reference number 2020/ETH03128) on 21 February 2021. This study was conducted in accordance with the Declaration of Helsinki, and informed written or verbal consent was obtained from all study participants. In cases of verbal consent, it was obtained according to a standard consent script and a documentation form that was approved by the South Western Sydney Local Health District Human Research Ethics Committee.

## 3. Results

**Figure 2:**
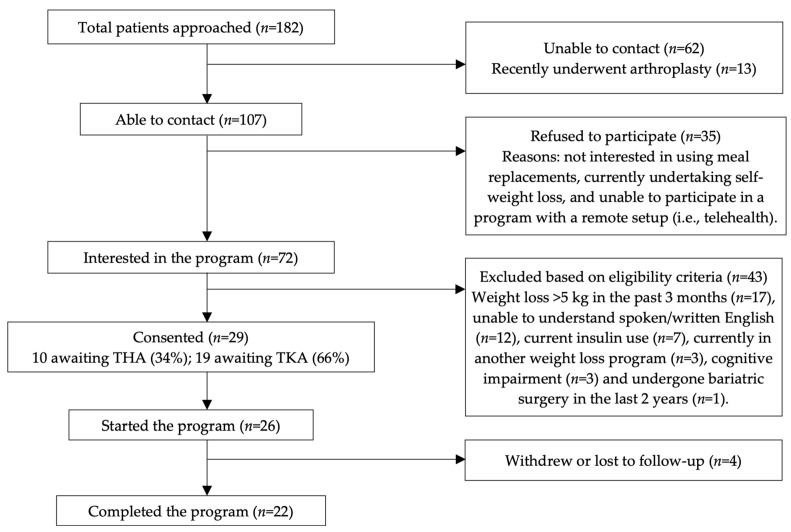
TREND flowchart of the participants recruitment and program completion.

A total of 182 patients were approached between January and April 2022, of whom 13 had recently undergone arthroplasty, 62 were unable to be contacted, and 35 out of the remaining 107 contacted declined to participate in the program (Figure 2). The remaining 72 patients who were interested in the program were screened for eligibility, of which 43 were excluded (Figure 2). Of the 29 who consented to participate in this study, 26 (89.7%) started the weight loss program and 22 (75.9%) completed the 12-week PMR plan. Two participants were lost to follow-up despite numerous attempts to contact them during the time of providing the second instalment of meal replacement shakes (i.e., before week 4). Additionally, two participants withdrew their consent, and it was confirmed during their dietitian appointment in week 4 that they were unable to adhere to the plan.

Baseline characteristics of the study participants are presented in Table 2. The participants were aged 63 (95% CI: 60.2, 65.8) years with a BMI of 39.4 (95% CI: 36.7, 42.2) kg/m^2^. 

### 3.1. Weight Loss

By the end of the 12-week PMR plan, 15/22 (68.2%) of the participants who started the program achieved at least 5% weight loss compared to baseline, with a mean weight loss of 6.1% (95% CI: 4.6, 7.5). As shown in Table 3, there was a statistically significant weight loss from baseline (104.1 kg; 95% CI: 94.9, 113.3) to 12 weeks follow-up (97.8 kg; 95% CI: 88.8, 106.9), with a paired difference of −6.3 kg (95% CI: −4.8, −7.7; *p* < 0.001). This resulted in a change in BMI by −2.4 kg/m^2^ (95% CI: −1.7, −3.0; *p* < 0.001). 

### 3.2. Acceptability, Adherence and Safety

Participants were provided with the 12-week supply of the meal replacements (168 sachets in total) in two or three instalments free of cost. All 22 participants who completed the program demonstrated acceptability and adherence to the 12-week PMR plan by the significant weight change along with enthusiasm to collect the meal replacements at different time-points over the study duration. Moreover, the remote nature of the PMR weight loss program was feasible and well accepted by all participants as those who completed the program were able to participate in the online education sessions and attended all of the telehealth appointments. The average acceptable cost that participants said they would pay for the low-calorie shakes was reported at A$69.5 per fortnight (28 sachets required per fortnight: A$2.5 per sachet). Most participants (18/22) who completed the 12-week PMR plan reported that they were willing to pay for the service if offered on a long-term basis after the arthroplasty. In terms of safety of the intervention, no adverse health related events or nutritional deficiencies were reported during the 12-week study duration, as well as after 12 weeks of intervention. Additionally, there appeared to be no major safety concerns among the people who were lost to follow-up based on a review of their electronic medical records.

### 3.3. Changes in Biochemical Profile, Questionnaire-Based Scores, and Lifestyle Behaviours

From baseline to 12 weeks, there were statistically significant reductions in HbA1c (with paired difference: −0.2%; 95% CI: −0.0, −0.4; *p* = 0.020) and low density lipoprotein (LDL) (with paired difference: −0.3 mmol/L; 95% CI: −0.1, −0.5; *p* = 0.004). However, changes in micronutrients or questionnaire-based scores were not observed during the 12 weeks (Table 3). As shown in Table 4, the readiness to change lifestyle behaviour questionnaire revealed the proportion of participants in action (Yes, and I have been doing so for less than 6 months) and maintenance (Yes, and I have been doing so for more than 6 months) phases in the readiness to change diet—“Do you eat less sugary foods and carbohydrates” increased significantly at 12 weeks (66.7% vs. 100%, *p* = 0.009). Similar changes were also observed in readiness to change physical activity—“Are you making yourself stronger?” (47.6% vs. 90.5%, *p* = 0.006) and readiness to change weight—“Are you trying to reach your best weight?” (47.6% vs. 95.2%, *p* = 0.001). 

## 4. Discussion

This study demonstrated that a 12-week PMR weight loss program was feasible and well accepted by individuals awaiting arthroplasty, despite being conducted remotely during COVID-19 restrictions and lockdowns. This program resulted in significant weight loss among people with obesity who were awaiting TKA or THA, with over half of participants achieving at least 5% weight loss. Associated benefits included lowering of HbA1c and LDL over 12 weeks and an increase in the proportion of people in the action and maintenance phases of the readiness to change diet, physical activity, and weight. 

The study findings demonstrate that significant weight loss (over 5%) is achievable following a 12-week PMR weight loss program for people awaiting TKA or THA. The weight loss of 6.3 kg achieved at 12 weeks in this study is comparable with that in a prior study (4.7 kg) utilising a 10-week PMR weight loss program [48]. Another study utilising a 12-week PMR plan among people with type 2 diabetes also reported a comparable weight loss of 5.7 kg at 12 weeks, which was retained in the long-term with an average weight loss of 7.1 kg and 4.2 kg at 12 and 24 months [28]. The prior study also reported a significant reduction in HbA1c following the 12-week PMR plan [28], which was also observed among the participants of this study. While these findings indicate that significant weight loss can be achieved with meal replacements, there is a need for longer duration future studies to evaluate the long-term effectiveness of the PMR weight loss program post-surgery among people undergoing arthroplasty. 

The effectiveness of meal replacements in achieving significant weight loss has also been established in a similar population. Following a 12-week PMR weight loss program prior to orthopaedic surgery, slightly higher weight loss was achieved among women with knee osteoarthritis (6.1% vs. 8.23%) [27] and among patients with chronic hip or knee osteoarthritis (6.3 kg vs. 7.56 kg) [49]. In comparison, a shorter-duration intensive weight loss therapy for 8 weeks with a low-energy liquid diet has been shown to result in even greater weight loss of 10.7 kg prior to TKA, which was retained one year after surgery [50]. The greater weight loss in these prior studies [49,50] may have led to greater improvements in biochemical measures including triglycerides and total cholesterol, apart from the LDL cholesterol observed in this study. Prior studies have also shown improvement in quality of life pre- and post-surgery [27,50], along with improvement in knee function one year post-surgery [50]. While there were no significant changes in index joint symptoms or quality of life, the broader benefits of the PMR program were demonstrated by positive behavioural changes, including the significant increases in the proportion of people in the action and maintenance phases of the readiness to change diet, physical activity, and weight. Moreover, nutritional deficiencies were not detected in participants undertaking the 12-week PMR plan, with no significant changes in biochemistry or micronutrients. This finding supports the adequacy of using meal replacements, which are fortified with vitamins and minerals, as part of clinician-supported weight loss programs to achieve micronutrient adequacy [51]. 

The overall weight change achieved in this study could be linked with the study being conducted at the time of the COVID-19 pandemic, where weight gain was often reported among the general population due to the change in lifestyle behaviours [52,53,54]. It is essential to note that the weight loss and significant reduction in BMI in this study was achieved in spite of people being restricted to staying at home and the decreased outdoor activity due to the COVID-19 restrictions at the time of the intervention. Moreover, the remote delivery of the intervention meant that the online contact was limited to three online group education sessions and three rounds of one-to-one telephone/telehealth support with a dietitian over 12 weeks. It is noteworthy that the weight loss was achieved without structured face-to-face visits in a population who may not be seeking weight loss as their primary goal, which suggests that remote delivery of weight loss and lifestyle interventions may be effective [55]. Further, it is well known that telehealth services may be more accessible to people with better computer literacy; thus, our program may not be appropriate for those who have few computer skills or limited access [56]. It is likely that the use of the structured PMR App in this study may have helped with adherence and motivation to continue with the weight loss program. As self-monitoring of weight, dietary intake and physical activity has been shown to be positively associated with weight loss, the use of the PMR App may have the potential to help maintain the weight loss in the long-term [57].

This study found that the 12-week PMR weight loss program was feasible and well accepted by people awaiting TKA or THA, with the participants signifying substantial willingness to pay for the meal replacements as well as the service on a long-term basis. This finding of the acceptability of a PMR weight loss program, along with the effectiveness of meal replacements in achieving significant weight loss, is consistent with prior studies conducted among diverse populations with overweight or obesity [48,58,59,60]. While people awaiting arthroplasty may not represent a population who are actively seeking help for weight loss [29], the PMR program in this study generated significant interest among those approached despite the intervention being delivered remotely during the COVID-19 restrictions. A high proportion of participants who consented to participate (*n* = 29) were able to start and complete the 12-week PMR plan (89.7% and 75.9%, respectively), which further demonstrate the feasibility of the PMR weight loss program. 

The main strength of this study is that it provides real-world evidence by including patients who were on a waiting list for TKA or THA in a high-volume public arthroplasty centre. Moreover, the remote delivery of the 12-week PMR weight loss program was well supported by the multidisciplinary team that included a dietitian, along with additional support provided through the structured PMR App. As such, the PMR weight loss program is easy to adapt and can be successfully replicated to achieve significant weight loss among patients awaiting TKA or THA. Furthermore, the remote delivery format of the PMR weight loss program has important implications for increasing access to weight loss interventions, particularly for people who may face barriers to attending in-person programs.

The study findings should be considered in light of some limitations. These include the absence of a control group or randomisation, as well as the short-term follow up period of 12 weeks. However, based on previous research in this patient population group [31], we are confident that minimal weight loss would have occurred amongst those not involved in the PMR weight loss program. The generalisability of the study findings is also limited by the small sample (*n* = 29) of participants awaiting TKA or THA recruited from a single public arthroplasty centre. Another limitation was the self-recorded weight collected from the participants over the study duration necessitated by COVID-19 social restrictions. However, the reliability of self-reported weight in our study is supported by the cross-validation with photographs of scale readings provided by the participants, along with logs of data entered in the PMR App and electronic medical records. Additionally, the congruence between the trends observed in self-reported weight and the improvements in objective markers, such as HbA1c and LDL, further corroborates the accuracy and reliability of the self-reported weight data. 

While participants in this study achieved a significant weight loss of 6.3 kg, a limitation of our study is the lack of detailed analysis regarding the composition of weight loss, including the percentage of fat mass and fat free mass as well as fluids lost, which would have provided valuable insights into the potential impact on surgical outcomes, infection prevention, rehabilitation programs, and trauma healing [61]. Another limitation is that post-surgical assessments were not possible due to surgery postponements consequent to COVID-19 disruptions to elective surgical lists. It should also be noted that the entire 12-week PMR weight loss program was delivered remotely via telehealth due to COVID-19 restrictions. Nonetheless, it can be posited that the program may be more effective if delivered face-to-face for some of the visits instead of a completely remote setup. Future research could address some of these limitations by conducting larger-scale trials with longer follow-up periods and comparison of the intervention delivered face-to-face or remotely in the post-pandemic era among other populations and settings. 

## 5. Conclusions

The study findings demonstrate that significant weight loss of over 5% is achievable for people with obesity awaiting arthroplasty following a 12-week PMR weight loss program. There were associated benefits including lowering of HbA1c and LDL over 12 weeks and an increase in the proportion of people in action and maintenance phases in readiness to change their diet, physical activity, and weight. Despite the intervention being conducted remotely during COVID-19 restrictions, this study’s findings indicate that the PMR weight loss program was feasible and well accepted by people awaiting TKA or THA, with many participants reporting willingness to pay for the service if offered on a long-term basis following the arthroplasty.

## Figures and Tables

**Table 1:** Summary of data collection measures at different timepoints during the study.

Variable/Outcome	Baseline	12 Weeks
Demographics (e.g., age, sex, education, ethnic background)	√	
Past medical history	√	
Weight and body mass index (BMI)	√	√
Medications (names, dose, and frequency of all medications currently prescribed)	√	√
Biochemical measures (blood sugar and insulin, lipid profile, liver function test, kidney function test, full blood counts and micronutrients)	√	√
Index joint symptoms (Oxford Knee and Hip Score) [40,41]	√	√
Quality of life using the EuroQol Five-Dimension Visual Analog Scale (EQ-5D VAS) [42]	√	√
Wellbeing using the World Health Organisation Five (WHO-5) Well-being Index [43]	√	√
Psychological distress using the Kessler Psychological Distress Scale (K10) [44]	√	√
Readiness to change lifestyle behaviour using a validated questionnaire based on the Transtheoretical (“stages of change”) questionnaire, which includes readiness to change physical activity (5 items), diet (7 items) and weight (1 item) [45,46]	√	√
International Physical Activity Questionnaire (IPAQ) Short Form 7 days [47]	√	√
Acceptability—adherence (count of meal replacements issued; weight change)		√
Willingness to pay (participants were asked to state their maximum willingness-to-pay for the low-calorie shakes per fortnight)	√	√
Safety—instances of acute kidney injury or nutritional deficiencies		√

**Table 2:** Baseline characteristics of the study participants.

Characteristics *	Mean (95% CI) or n (%)
*Sociodemographic Characteristics*	
Age (in years)	63.0 (60.2, 65.8)
Sex (female)	19 (65.5%)
Employment (in paid employment)	7 (24.1%)
Country of birth (Australia)	12 (41.4%)
*Comorbidities and Clinical Characteristics*	
Weight (in kg)	105.8 (98.2, 113.3)
Body mass index (BMI) (in kg/m^2^)	39.4 (36.7, 42.2)
Type 2 diabetes	9 (31.0%)
Hypertension	16 (55.2%)
Dyslipidaemia	11 (37.9%)
Obstructive sleep apnoea	7 (24.1%)

* Total participants = 29, of which 10 were awaiting THA and 19 were awaiting TKA.

**Table 3:** Changes in clinical measures, questionnaire-based scores, and biochemical measures.

Variable Mean (95% CI) or n (%)	Baseline (*n* = 29)	12 Weeks (*n* = 22)	Paired Difference Mean (95% CI)	*p*-Value
Weight (in kg)	104.1 (94.9, 113.3)	97.8 (88.8, 106.9)	−6.3 (−4.8, −7.7)	<0.001
BMI (in kg/m^2^)	38.6 (35.8, 41.4)	36.2 (33.6, 38.9)	−2.4 (−1.7, −3.0)	<0.001
**Questionnaire-based scores**				
Oxford Knee Score ^a^	16.2 (11.7, 20.7)	17.9 (12.1, 23.7)	1.7 (6.5, −3.1)	0.472
Oxford Hip Score ^b^	13.3 (7.5, 19.2)	11.2 (4.7, 17.7)	−2.2 (1.9, −6.3)	0.234
EQ-5D VAS	48.3 (39.9, 56.7)	53.3 (45.3, 61.6)	5.0 (14.5, −4.5)	0.284
WHO-5 Well-being Index	42.8 (32.5, 53.2)	46.4 (35.9, 56.0)	3.0 (12.2, −6.1)	0.493
K10	22.7 (18.2, 27.2)	22.1 (17.8, 26.3)	−0.7 (1.8, −3.1)	0.585
**Biochemical profile ^c^**				
*Measures of blood sugar and insulin*				
HbA1c (in %)	6.2 (5.8, 6.6)	6.0 (5.7, 6.3)	−0.2 (−0.0, −0.4)	0.020
Fasting plasma glucose (in mmol/L)	6.3 (5.3, 7.4)	5.9 (5.2, 6.7)	−0.4 (0.3, −1.0)	0.462
Fasting insulin (in mU/L)	21.1 (16.2, 26.0)	19.7 (15.0, 24.4)	−1.4 (1.4, −4.2)	0.494
*Lipid profile*				
Total cholesterol (in mmol/L)	4.7 (4.1, 5.1)	4.4 (4.1, 4.9)	−0.2 (0.2, −0.5)	0.290
Triglyceride (in mmol/L)	1.8 (1.5, 2.1)	1.7 (1.4, 2.0)	−0.1 (0.2, −0.4)	0.498
HDL (in mmol/L)	1.4 (1.1, 1.7)	1.5 (1.1, 1.9)	0.1 (0.4, −0.2)	0.472
LDL (in mmol/L)	2.8 (1.9, 3.8)	2.5 (1.4, 3.6)	−0.3 (−0.1, −0.5)	0.004
*Biochemistry and micronutrients*				
Creatinine (in μmol/L)	75.8 (68.0, 83.6)	70.1 (60.4, 80.0)	−5.59 (3.9, −15.1)	0.235
eGFR (in ml/min/1.73 m^2^)	79.1 (73.4, 84.7)	78.0 (69.1, 86.9)	−1.1 (5.8, −8.0)	0.364
Vitamin D (in nmol/L)	61.4 (51.6, 71.1)	74.1 (49.4, 98.7)	12.7 (35.6, −10.3)	0.261
Adjusted calcium (in mmol/L)	2.4 (2.4, 2.5)	2.4 (2.4, 2.5)	0.0 (0.0, −0.1)	0.206
Magnesium (in mmol/L)	0.9 (0.8, 0.9)	0.8 (0.8, 0.9)	−0.1 (0.0, −0.1)	0.085
Phosphate (in mmol/L)	1.3 (0.9, 1.7)	1.2 (1.1, 1.2)	−0.2 (0.2, −0.5)	0.602
Iron (in μmol/L)	15.2 (12.9, 17.5)	15.9 (10.9, 21.0)	0.8 (5.2, −3.7)	0.823
Vitamin B12 (in pmol/L)	275.2 (218.2, 332.1)	270.7 (230.8, 310.6)	−4.5 (37.0, −46.0)	0.999
Folate (in nmol/L)	29.8 (24.8, 34.9)	27.2 (23.1, 31.4)	−2.6 (0.6, −5.8)	0.107
*Liver function test*				
ALP (in IU/L)	81.9 (68.0, 95.7)	83.7 (73.3, 94.1)	1.8 (10.7, −7.1)	0.677
GGT (in IU/L)	36.7 (29.1, 44.3)	34.0 (25.9, 42.1)	−2.7 (2.4, −7.8)	0.455
ALT (in IU/L)	35.8 (22.3, 49.4)	31.3 (24.1, 38.5)	−4.5 (11.8, −20.9)	0.767
AST (in IU/L)	26.7 (20.4, 33.1)	23.2 (20.2, 26.2)	−3.5 (1.1, −8.3)	0.177
*Serum based liver fibrosis score*				
FiB-4 Score	1.2 (0.8, 1.6)	1.1 (0.8, 1.3)	−0.1 (0.1, −0.3)	0.758
APRI Score	0.3 (0.2, 0.4)	0.2 (0.2, 0.3)	−0.1 (0.0, −0.1)	0.277

Abbreviations: ALP: alkaline phosphatase; ALT: alanine transaminase; APRI: AST to platelet ratio index; AST: aspartate aminotransferase; BMI: body mass index; CI: confidence interval; eGFR: estimated glomerular filtration rate; EQ-5D VAS: EuroQol Five-Dimension Visual Analog Scale; FiB-4: Fibrosis-4 Index for Liver Fibrosis; GGT: gamma-glutamyl transferase; HbA1c: haemoglobin A1c; HDL: high density lipoprotein; K10: Kessler Psychological Distress Scale; LDL: low density lipoprotein; WHO: World Health Organization. ^a^ Only 14 participants completed the paired Oxford Knee Score. ^b^ Only 6 participants completed the paired Oxford Hip Score. ^c^ The total number does not add up to 22 participants.

**Table 4:** Changes in readiness to change to healthy lifestyle and physical activity.

Variable n (%)	Baseline	12 Weeks	*p*-Value
**Readiness to change lifestyle behaviour (n and %) for action and maintenance stage**			
*Dietary change—readiness to change diet*			
Do drink water and other non-sugary drinks instead of sugary drinks/fruit juice	18 (85.7%)	20 (95.2%)	0.606
Do you eat at least four or more servings of vegetables daily	9 (42.9%)	14 (66.7%)	0.215
Do you eat at least three different proteins foods every 1–2 days	17 (80.9%)	19 (90.5%)	0.662
Do you eat less fat overall?	13 (61.9%)	19 (90.5%)	0.067
Have you reduced the amount of food you eat at each sitting	16 (81.0%)	20 (95.5%)	0.183
Do you eat more foods with fibre	15 (71.4%)	20 (95.2%)	0.093
Do you eat less sugary foods and carbohydrates	14 (66.7%)	21 (100.0%)	0.009
*Physical activity—readiness to change physical activity*			
Are you making yourself stronger?	10 (47.6%)	19 (90.5%)	0.006
Do you plan more activity in your weekday?	7 (33.3%)	11 (52.4%)	0.350
Do you plan more activity on weekends?	7 (23.8%)	13 (61.9%)	0.121
Have you increased the number of steps you take each day?	9 (42.9%)	11 (52.4%)	0.758
Have you reduced the amount of time you spend sitting?	7 (33.3%)	12 (57.1%)	0.215
*Weight—readiness to change weight*			
Are you trying to reach your best weight?	10 (47.6%)	20 (95.2%)	0.001
**Physical activity IPAQ—Short Form 7 days ^a^**			
Have you participated in vigorous activity in the past 7 days	4 (20.0%)	2 (10.0%)	0.661
Have you participated in moderate activity in the past 7 days	5 (25.0%)	7 (35.0%)	0.731
Have you walked more than 10 min at a time in the last 7 days	17 (85.0%)	17 (85.0%)	1.000
IPAQ: MET-min per week	338.3 (1072.5) ^b^	179.0 (1388.3) ^b^	0.246

^a^ Only 20/22 participants completed the IPAQ Short Form. ^b^ Median (IQR) used. IPAQ: International Physical Activity Questionnaire.

## Data Availability

The data used to support the findings of this study are available from the corresponding author upon request.

## Supplementary Materials

The following supporting information can be downloaded at: https://www.mdpi.com/article/10.3390/jcm13113227/s1, Table S1: TREND statement checklist.
